# Myocarditis and Drug Rash With Eosinophilia and Systemic Symptoms Syndrome: A Deadly Combination

**DOI:** 10.7759/cureus.13496

**Published:** 2021-02-22

**Authors:** Angel C De La Cruz, Shoaib Ashraf, Nikee Shrestha, Muhammad Saad

**Affiliations:** 1 Internal Medicine, Bronx Care Health System, Bronx, USA; 2 General Internal Medicine, Bronx Care Health System, Bronx, USA

**Keywords:** myocarditis, dress syndrome, lymphadenopathy, echocardiography, amoxicillin

## Abstract

DRESS syndrome (Drug Rash with Eosinophilia and Systemic Symptoms) is a severe delayed type IV hypersensitivity drug reaction by T helper cell 2 (Th2) and Interleukin 5 (IL-5) resulting in activation of eosinophils. It is mostly reported with antiepileptic drugs (AEDs), antibiotics, and allopurinol. Here, we present the second case of myocarditis secondary to DRESS syndrome caused by amoxicillin. Most of the case reports present with cross-reactivity among the anticonvulsants and beta-lactams, which is also rarely been reported. Amoxicillin could reactivate human herpesvirus 6 (HHV 6) and Epstein-Barr virus (EBV) with a presentation similar to DRESS syndrome, but our patient was neither taking the anticonvulsants nor have any viral infection in the recent past. His RegiSCAR score was 6, consistent with definite DRESS syndrome. Management includes identification and prompt withdrawal of the offending drug and supportive care for patients without severe organ involvement and systemic corticosteroids for patients with severe organ involvement.

## Introduction

DRESS syndrome (Drug Rash with Eosinophilia and Systemic Symptoms), also known as drug-induced hypersensitivity syndrome, is a severe idiosyncratic drug reaction. It is a delayed-type IV hypersensitivity reaction by T helper cell 2 (Th2), and Interleukin 5 (IL-5) resulting in activation of eosinophils [1]. DRESS is most commonly reported with antiepileptic drugs (AEDs), antibiotics, and allopurinol [2]. It affects the skin and various internal organs anywhere from two to eight weeks after exposure to a culprit medication [3]. DRESS syndrome typically presents with widespread skin rash, fever, lymphadenopathy, hematologic abnormalities (eosinophilia, atypical lymphocytosis), and end-organ damage, most commonly to the kidneys, lungs, and liver. We present the second case of myocarditis secondary to DRESS syndrome caused by amoxicillin. Cardiac involvement varies considerably between 4% and 21%, which can be fatal and therefore requires prompt diagnosis and treatment [4]. RegiSCAR scoring system helps clinicians in confirming or excluding the diagnosis of DRESS syndrome [5]. Management includes identification and prompt withdrawal of the offending drug and supportive care for patients without severe organ involvement. For patients with severe organ involvement, treatment with systemic corticosteroids is suggested but has not been evaluated in randomized trials [6].

## Case presentation

A 33-year-old Hispanic man, with a recent dental procedure presented to our hospital complaining of right-sided neck pain and swelling for three days. He also complained of fever, chills, headache, and non-pruritic deep red blanching macular eruption on the trunk and extremities which extends to palms and soles. He is from the Dominican Republic, immigrated to the United States three months ago. A week before the presentation, the patient had a dental procedure for which he was treated with the amoxicillin antibiotic and a nonsteroidal anti-inflammatory drug (NSAID) for three days. Later on, he developed fever, malaise, headache, and macular eruption on the trunk and extremities. 

**Figure 1:**
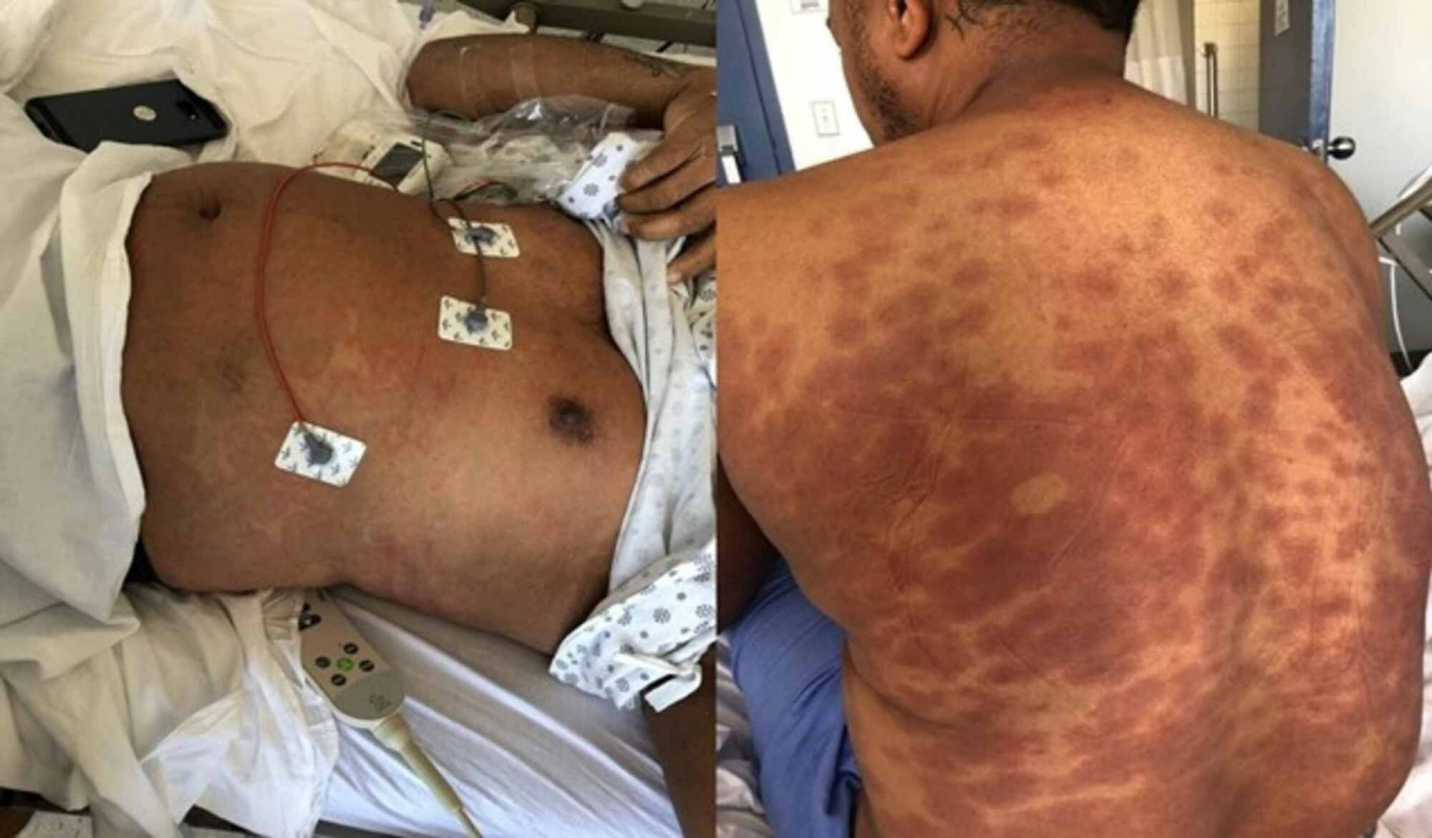
Cutaneous manifestations in patient Erythematous macules and papules on the entire body

In the emergency department, he was ill-appearing and was in moderate distress. His vital signs on admission included a body temperature of 102.3 °F, blood pressure of 122/76 mmHg, heart rate of 120 beats/min, respiratory rate of 15 breaths/minute, and oxygen saturation of 99% on room air. The patient’s cardiovascular examination revealed a regular heart rate without audible heart murmurs or an elevated jugular venous pressure. He had no peripheral edema of the lower extremities with normal peripheral pulses. The respiratory examination revealed normal vesicular breath sounds, no wheezing. His skin showed non-pruritic deep red blanching macular eruption on the trunk and extremities which extends to palms and soles. The rest of the physical examination was within normal limits (Figure 1). The Nikolsky sign and Koplik spots were negative.

Laboratory investigations showed a white blood cell (WBC) count of 13.6 × 10^3^/µL, with 2.8% eosinophils and 81.3% neutrophils, and a platelet count of 345 ×10^3^/μL. Blood chemistry results showed blood urea nitrogen (BUN) of 10 mg/dL, serum creatinine of 0.9 mg/dL, aspartate aminotransaminase (AST) of 63 U/mL, and alanine aminotransferase (ALT) of 66 U/mL.

Serological tests for the presence of hepatitis B surface antigen (HbsAg), surface antibody (anti-HBs), total hepatitis B core antibody (anti-HBc), hepatitis C antibody and its polymerase chain reaction (PCR) test, varicella-zoster IgM antibody, and cytomegalovirus IgM antibody were all negative, indicating that these viruses were unlikely to be the cause of his condition. Serological testing for Epstein-Barr virus (EBV) was negative for EBV DNA, serum heterophile agglutination, EBV viral capsid antigen IgG, and EBV viral capsid antigen IgM. Serological testing for herpes simplex virus (HSV) 1 and 2 DNA and IgM screen was negative but HSV Ab types 1 and 2 were positive indicating a history of HSV infection. Serological testing for measles Ab IgM (EIA) was negative but measles (rubeola) antibodies, IgG was positive, indicating a history of rubeola infection. The results of serological tests for the presence of cytomegalovirus (CMV) IgM (EIA), varicella-zoster virus (VZV) IgM, human parvovirus B19 IgM, and human immunodeficiency virus (HIV) antibody were negative.

**Figure 2:**
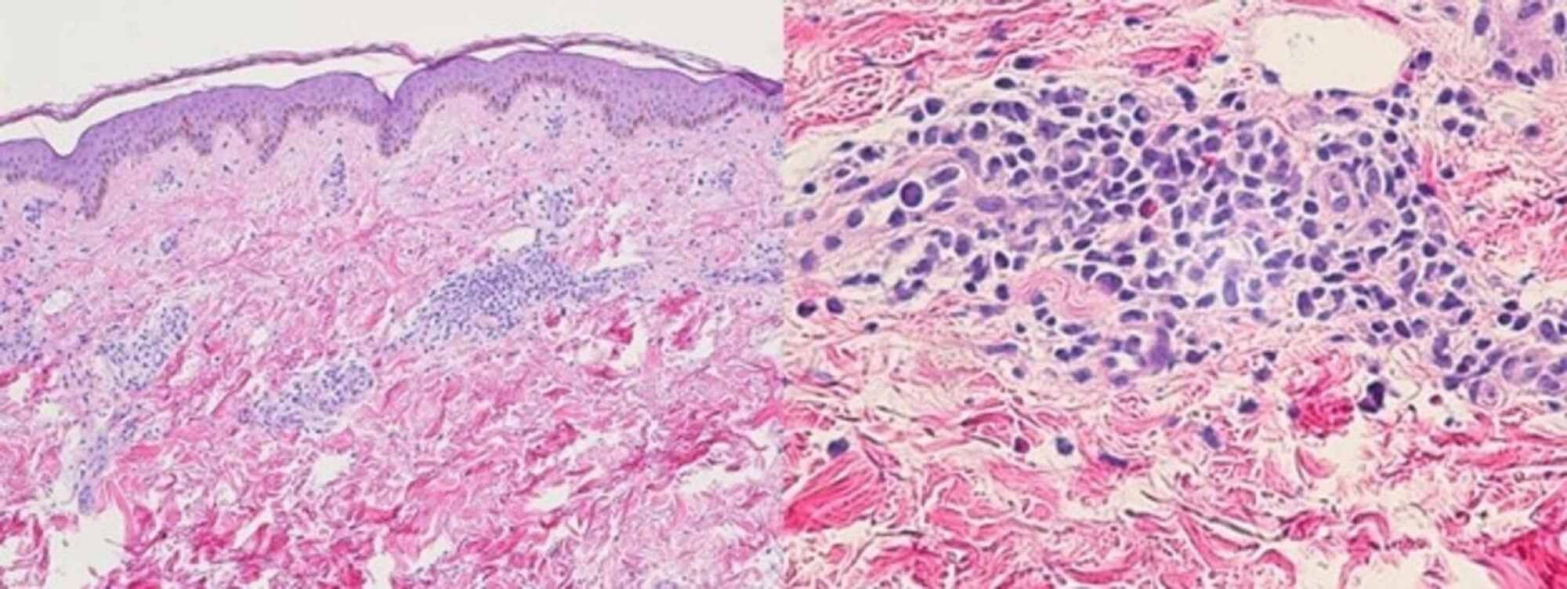
Skin biopsy and histopathology of the patient Skin biopsy showed superficial perivascular dermatitis with lymphocyte and eosinophils infiltrate

Blood cultures, aerobic throat culture, and blood fungal culture resulted in normal; serologic tests for an acute viral infection by adenovirus, human rhinovirus, Influenza (A and B), Influenza A virus H1/H3 subtype by PCR, human metapneumovirus (hMPV), human respiratory syncytial virus (RSV A and B), and human parainfluenza (1, 2, 3) resulted negative according to the cutoffs of our laboratory. Among autoantibodies, rheumatoid factor, antinuclear antibody (ANA), antibody to antiscleroderma-70, anti-cyclic citrullinated antibody, anti-SM, antistreptolysin O, myeloperoxidase, proteinase-3 Ab, anti-Ro/SSA, and anti-La/SSB antibodies, anti-Jo-1 antibody, anti-Sm, anti-RNP antibodies, anti-DNA antibodies, and lupus anticoagulants were negative. Tests for syphilis, including the rapid plasma reagin (RPR) test were negative. Skin biopsy and histopathology showed superficial perivascular dermatitis with lymphocyte and eosinophils infiltrates (Figure 2). The changes are consistent with hypersensitivity reactions such as drugs or orthropodocytes.

**Figure 3:**
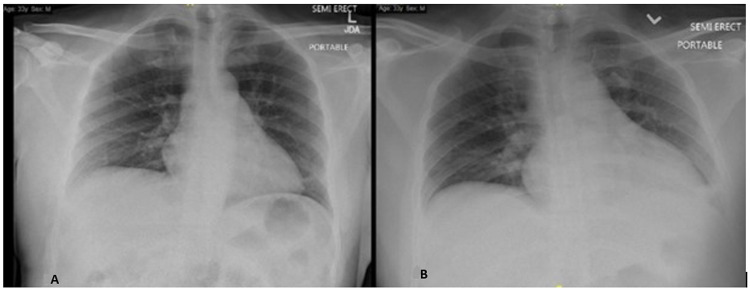
Chest X-ray of the patient (A) chest X-ray showed no evidence of active chest disease; (B) chest x-ray demonstrating cardiomegaly, left basilar infiltrate versus atelectasis

The first chest X-ray showed no evidence of active chest disease (Figure 3A). Repeat chest x-ray on the floor demonstrating cardiomegaly, left basilar infiltrate versus atelectasis (Figure 3B).

**Figure 4:**
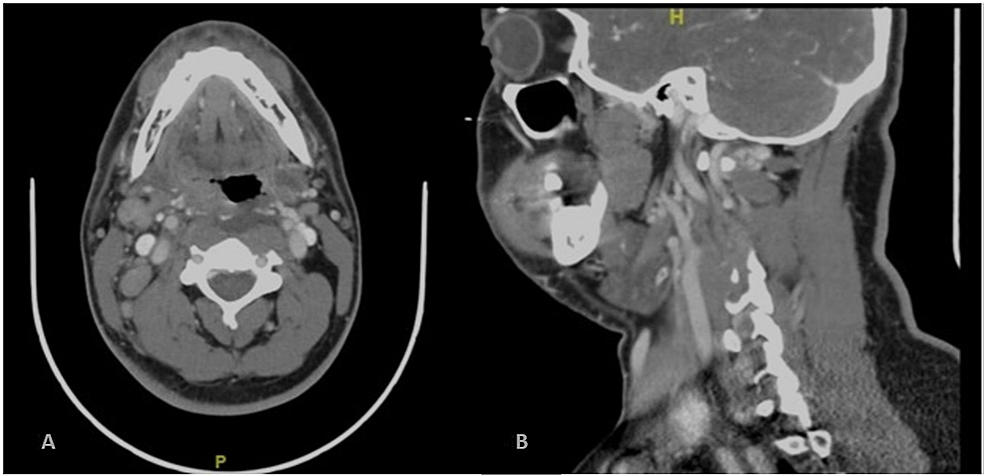
Neck computed tomography (A) Axial section showed a retropharyngeal abscess and enlarged right posterior triangle lymph nodes; (B) coronal image showed a retropharyngeal abscess

A head computed tomography (CT) scan showed no acute infarct, intracranial bleeding, or mass effect. A neck CT scan showed a retropharyngeal abscess and enlarged right posterior triangle lymph nodes (Figure 4).

The patient was initially started on ceftriaxone and clindamycin for the retropharyngeal abscess but due to persistent fever, high erythrocyte sedimentation rate (ESR) 90.0 mm/hour, and C-reactive protein (CRP) 285.84 mg/L antibiotics were changed to vancomycin, aztreonam, metronidazole, and doxycycline for broader coverage.

Four days after admission, the rash became more confluent with annular and polycyclic lesions with dusky centers, but fever persisted. We used the RegiSCAR scoring system that grades our patient was definite DRESS syndrome (Table 1). Five days after admission, he developed intermittent chest discomfort and malaise with persistent fever despite antibiotics. Electrocardiography (ECG) showed normal sinus rhythm with 88 beats/minute, first-degree atrioventricular (AV) block, and moderate voltage criteria for left ventricular hypertrophy.

**Table 1:** RegiSCAR scoring system

According to the original publication, cut-off points include: <2 No DRESS, 2-3 Possible, 4-5 Probable, ≥6 Definite		
Fever ≥38.5 °C	Yes	0
Enlarged lymph nodes ≥2 sites, ≥1 cm	Yes	1
Atypical lymphocytes	No	0
Eosinophilia	No	0
Skin rash	Yes	
- Extend >50%	Yes	1
- At least two of the following: edema, infiltration, purpura, scaling	Yes	1
- Biopsy suggesting DRESS	Yes	0
Internal organ involved	≥2 organs	2
Resolution delay	>15 days	0
At least three biological investigations done and negative to exclude the alternative diagnosis	Yes	1
RegiSCAR score		
Assessment	Definite DRESS	6

**Figure 5:**
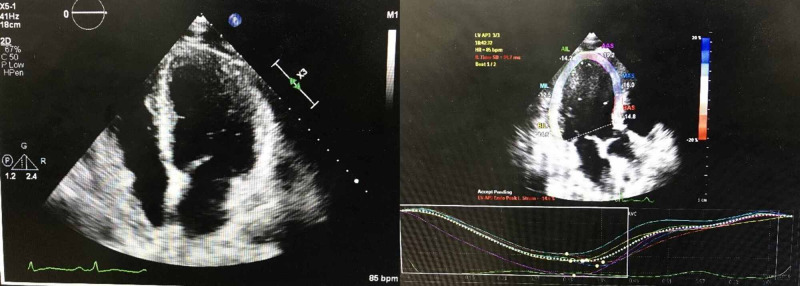
Echocardiography Concentric remodeling with global hypokinesis, grade 2 diastolic dysfunction, and left ventricular ejection fraction of 49.29%

The patient was found to have troponin of 447 ng/ml and repeat 562 ng/ml with a delta of 115 ng/ml, N-terminal pro-B-type Natriuretic Peptide (Pro BNP) was 4835 pg/ml, and CRP 285.84 mg/L. Echocardiography following admission showed a concentric remodeling with global hypokinesis and grade 2 diastolic dysfunction, and pericardial effusion was seen without echocardiographic signs of tamponade. Left ventricular systolic function was diffusely reduced with left ventricular ejection fraction (LVEF) of 49.29% (Figure 5), right ventricular systolic pressure 41.58 mmHg consistent with mildly elevated pulmonary artery pressure. Endomyocardial biopsy was discussed with the patient but he refused the biopsy.

He was started on a standard heart failure regimen consisting of carvedilol, lisinopril but the diuretic was not started as the patient was euvolemic. He was placed on intravenous immune globulin (IVIG) and began triamcinolone 0.1% ointment applied to his whole body. High-dose glucocorticoids were avoided because of myocarditis. Repeat neck CT scan showed resolution of a retropharyngeal abscess, blood cultures came back negative, so antibiotics were stopped. Troponin and N-terminal pro-B-type natriuretic peptide (BNP) levels came to normal limits. Repeat echocardiogram showed improvement in ejection fraction (EF). He slowly improved, became afebrile with a gradual central clearing of the rashes and clinical improvement.

## Discussion

DRESS is a distinct type of drug-induced hypersensitivity syndrome caused by a wide variety of drugs, although it is most commonly associated with anticonvulsants and antibiotics [6]. It carries about 10% mortality, with an incidence that ranges from 1 in 1000 to 10,000 drug exposures [6,7]. Most cases of DRESS occur in adults, and the female to male ratio is around 1:1.

The pathogenesis of this syndrome is unclear. Many hypotheses have been proposed, including genetic mutations involving deficient drug metabolism, an immunologic mechanism involving activation of drug-specific T cells, reactivation of herpesviruses-HHV6, CMV, and EBV, and many more. Anticonvulsant drugs such as phenytoin, carbamazepine, phenobarbital, and sulfonamides such as dapsone and sulfasalazine are the most common agents known to cause DRESS. During the initial phase of the syndrome, there is an expansion of regulatory T cells as well as cytotoxic and helper T cells. These cells will cross-react with the offending drug, and the activated cytotoxic T cells will cause tissue damage through secretion of interferon-gamma, interleukin-5, and tumor necrosis factor-alpha. Interleukin-5 will also mediate the eosinophilic infiltrate in the involved tissues.

Genetic deficiencies causing a deficiency in enzymes involved in drug catabolism have also been reported. Because of that, the buildup of drug metabolites can occur. These metabolites can bind to macromolecules inducing cell death in the involved organs. It has been reported that individuals with HLA-B1502, HLA-B1508, and HLA-B1502 are at increased risk of developing DRESS [8-10]. Generally, clinical signs and symptoms begin within two to six weeks after drug intake and include fever preceding various cutaneous manifestations - most commonly morbilliform rash and systemic findings. The most common systemic findings involve lymphatic, hematologic, and hepatic systems followed by renal, pulmonary, and cardiac systems. Patients may present with fever, skin rash, facial edema, extremity swelling, abdominal pain, chest pain, cervical lymphadenopathy, eosinophilia, atypical lymphocytosis, and multi-organ failure [11]. Several diagnostic criteria have been used for the diagnosis of DRESS, such as RegiSCAR and J-SCAR. The severity of DRESS depends upon the systemic involvement, which can result in multi-organ failure.

To the best of our understanding, we report the second case of amoxicillin-induced DRESS syndrome [12]. Potentially, amoxicillin can, directly and indirectly, cause DRESS syndrome. Multiple case reports of amoxicillin triggering DRESS syndrome through reactivation of HHV 6 and EBV [13,14] and through cross-reactivity to carbamazepine, sulfasalazine, and benzylpenicillin [7,15,16], have been reported. Nevertheless, DRESS syndrome directly caused by amoxicillin has been previously reported only once. In regards to the cases related to amoxicillin, few observations are notably in our case, the fact that the symptoms of DRESS flared within around five days later after the introduction of the antibiotic instead of a universally accepted time frame of two weeks to two months after the introduction of culprit drugs. A rash that appeared within two to five days after amoxicillin treatment is also observed in many related case reports [15,17,18]. Our patient neither had any active viral infection like HHV 6 and EBV nor was taking any medications that commonly are considered being causative agents for DRESS syndrome except amoxicillin. We believe that DRESS would obviously be related to amoxicillin in view of these arguments: (1) a clear-cut temporal relationship between the administration of amoxicillin and the symptoms onset (five days, typically two to five days [15,17,18]); (2) remission of the symptomatological pattern after the withdrawal of this drug and introduction of IVIG therapy and topical steroids; (3) the association of different symptoms evoking a clinical picture of DRESS syndrome. RegiSCAR scoring system grades our patient was probable DRESS syndrome (Table 1). There is no available gold standard for diagnosis of DRESS syndrome, but the RegiSCAR scoring system helps clinicians in the probability of diagnosis and inclusion criteria for hospitalization for the management of patients [19]. Our patient met six of seven RegiSCAR criteria.

Cardiac involvement in DRESS syndrome has been reported in only 4% to 21 % of cases of DRESS and it usually presents with myocarditis. Patients can present with no symptoms or can present with chest pain, dyspnea, and hypotension. Cardiomegaly and pleural effusions can be seen on the chest radiograph. EKG changes of ST and T wave changes and arrhythmias can be found, and decreased ejection fraction in echocardiogram can be seen. In addition, patients can present with elevation of cardiac enzymes. It is a very intriguing fact that there are two described forms of myocarditis in DRESS: hypersensitivity and acute necrotizing eosinophilic myocarditis (ANEM). ANEM is associated with more than 50% mortality, which is one of the reasons why this topic is important [20]. A definitive diagnosis is made by a cardiac biopsy. Management involves the removal of the offending drug. Beta-blockers and renin-angiotensin-aldosterone system modulators can also be used. Given the fact that DRESS can present with variable manifestations- with or without eosinophilia, with a variety of skin manifestations and a vast range of systemic involvement, one should have a high suspicion for diagnosis, especially with the fact that mortality in DRESS syndrome is around 10% with hepatic and cardiac involvement mortality rate increase significantly. 

For the most part, patients with DRESS recover entirely after about one month after the offending drug is withdrawn. About 10% of patients may develop complications that can include renal failure and the development of an autoimmune disease, such as Graves’ disease and diabetes mellitus type 1. Based on our experience, we recommend patient education and counseling about myocarditis, congestive cardiac failure, and pericarditis along with routine screens for cardiac enzymes in patients with DRESS. It is also essential to follow-up patients with signs and symptoms of heart failure with an echocardiogram and assesses them clinically, especially in the first six months after the DRESS syndrome because cardiac involvement can be quickly fatal.

Amoxicillin is believed to result in DRESS syndrome, but how amoxicillin can trigger DRESS syndrome is a topic of debate. Better knowledge of DRESS may contribute to understanding pathophysiology, improve the diagnosis and management of this syndrome in clinical practice.

## Conclusions

DRESS is a complex clinical syndrome associated with multiple medications and may involve many organs. Myocarditis is an uncommon complication of DRESS. When suspected, immediate action should be taken because it increases mortality significantly. In this document, we described a unique case of DRESS syndrome due to amoxicillin confirmed with a skin biopsy, clinical presentation, and RegiSCAR scoring system. The patient developed myocarditis, which makes this case to be even more unique. We strongly recommend patient and healthcare personnel education about the possible presentations of myocarditis along with routine screens for cardiac enzymes in patients with DRESS. Early identification of myocarditis and removal of the offending agent is key to reduce mortality in patients with DRESS and myocarditis. We highlight the importance of a structured approach to evaluate and investigate DRESS syndrome.

## References

[1] Damsky WE, Vesely MD, Lee AI (2019). Drug-induced hypersensitivity syndrome with myocardial involvement treated with tofacitinib. JAAD case reports.

[2] Kardaun SH, Sekula P, Valeyrie-Allanore L (2013). Drug reaction with eosinophilia and systemic symptoms (DRESS): an original multisystem adverse drug reaction. Results from the prospective RegiSCAR study. Br J Dermatol.

[3] Choudhary S, McLeod M, Torchia D, Romanelli P (2013). Drug reaction with eosinophilia and systemic symptoms (DRESS) syndrome. J Clin Aesthet Dermatol.

[4] Thongsri T, Chularojanamontri L, Pichler WJ (2017). Cardiac involvement in DRESS syndrome. Asian Pac J Allergy Immunol.

[5] Pannu AK, Saroch A (2017). Diagnostic criteria for drug rash and eosinophilia with systemic symptoms. J Family Med Prim Care.

[6] Cacoub P, Musette P, Descamps V, Meyer O, Speirs C, Finzi L, Roujeau JC (2011). The DRESS syndrome: a literature review. Am J Med.

[7] Watts TJ, Li PH, Haque R (2018). DRESS syndrome due to benzylpenicillin with cross-reactivity to amoxicillin. J Allergy Clin Immunol Pract.

[8] Choquet-Kastylevsky G, Intrator L, Chenal C, Bocquet H, Revuz J, Roujeau JC (1998). Increased levels of interleukin 5 are associated with the generation of eosinophilia in drug-induced hypersensitivity syndrome. Br J Dermatol.

[9] Chung WH, Hung SI, Hong HS (2004). Medical genetics: a marker for Stevens-Johnson syndrome. Nature.

[10] Hung SI, Chung WH, Liou LB (2005). HLA-B*5801 allele as a genetic marker for severe cutaneous adverse reactions caused by allopurinol. Proc Natl Acad Sci U S A.

[11] Karakayali B, Yazar AS, Cakir D (2017). Drug Reaction with Eosinophilia and Systemic Symptoms (DRESS) syndrome associated with cefotaxime and clindamycin use in a 6 year-old boy: a case report. Pan Afr Med J.

[12] Yu MK, Yu MC, Lee F (2006). Association of DRESS syndrome with chylous ascites. Nephrol Dial Transplant.

[13] Mardivirin L, Valeyrie-Allanore L, Branlant-Redon E (2010). Amoxicillin-induced flare in patients with DRESS (Drug Reaction with Eosinophilia and Systemic Symptoms): report of seven cases and demonstration of a direct effect of amoxicillin on Human Herpesvirus 6 replication in vitro. Eur J Dermatol.

[14] Kano Y, Inaoka M, Sakuma K, Shiohara T (2005). Virus reactivation and intravenous immunoglobulin (IVIG) therapy of drug-induced hypersensitivity syndrome. Toxicology.

[15] Bahat G, Celik HG, Tufan F, Saka B (2010). Drug rash with eosinophilia and systemic symptoms syndrome induced by sulfasalazine. Joint Bone Spine.

[16] Aouam K, Fredj Nadia B, Amel C, Naceur B (2010). Amoxicillin-induced hypersensitivity after DRESS To carbamazepine. World Allergy Organ J.

[17] Chadli Z, Ben Fredj N, Youssef M, Chaabane A, Boughattas NA, Zili JE, Aouam K (2016). The rest of the story of the patient described in the letter to the editors: 'Hypersensitivity to amoxicillin after… (DRESS) to carbamazepine…: a possible co-sensitization'. Br J Clin Pharmacol.

[18] Michel F, Navellou J-C, Ferraud D, Toussirot E, Wendling D (2005). DRESS syndrome in a patient on sulfasalazine for rheumatoid arthritis. Joint Bone Spine.

[19] Castellazzi ML, Esposito S, Claut LE, Daccò V, Colombo C (2018). Drug reaction with eosinophilia and systemic symptoms (DRESS) syndrome in two young children: the importance of an early diagnosis. Ital J Pediatr.

[20] Hagiwara H, Fukushima A, Iwano H, Anzai T (2018). Refractory cardiac myocarditis associated with drug rash with eosinophilia and systemic symptoms syndrome due to anti-bipolar disorder drugs: a case report. Eur Heart J Case Rep.

